# Individualized Ophthalmic Exoplants by Means of Reverse Engineering and 3D Printing Technologies for Treating High Myopia Complications with Macular Buckles

**DOI:** 10.3390/biomimetics5040054

**Published:** 2020-10-22

**Authors:** George Pappas, Nectarios Vidakis, Markos Petousis, Athena Maniadi

**Affiliations:** 1 Ophthalmology Department, General Hospital Venizeleio, 44 Knossou Avenue, 71409 Heraklion, Greece; retinacrete@gmail.com; 2 Mechanical Engineering Department, Hellenic Mediterranean University, Estavromenos, 71004 Heraklion, Greece; vidakis@hmu.gr; 3 Department of Materials Science and Technology, University of Crete, Vassilika, Voutes, 70013 Heraklion, Greece; maniadi@materials.uoc.gr

**Keywords:** myopic traction maculopathy, 3D printing, reverse engineering, biomaterials, pattern recognition, implant

## Abstract

Myopic macular foveoschisis maculopathy is an eye disease that is treated, in most cases, with surgical intervention, in which a macular buckle is applied to restore eye anatomy and functionality. A macular buckle is a type of exoplant that comes in various designs and sizes. Often, they are difficult to apply or they do not fit properly in the eye geometry since they have a generic form. In this work, the effort to develop the most suitable tailor-made macular buckle for each individual patient for treating myopic traction maculopathy is studied. Pattern recognition techniques are applied to the patient’s Computed Tomography (CT) data to develop the exact 3D geometry of the eye. Using this 3D geometry, the trajectory of the buckle is fitted and the buckle is formed, which is then 3D-printed with biocompatible polymer materials. It is expected that the power of technology will be used to activate the most precise approach for each individual patient. Considering the possible complications and technical difficulties of other surgical methods, the customized macular buckle is an appropriate, easy-to-use, and most precise piece of medical equipment for the treatment of myopic traction maculopathy.

## 1. Introduction

Myopic patients can develop a rare complication called Macular Foveoschisis (MF), and this can lead to retinal detachment, a potentially threatening condition for eyesight [1,2]. With the advancement of Optical Coherence Tomography (OCT) technology, the medical society has managed to learn more regarding this pathology [3]. OCT describes the structural changes developing in various levels of the retina and provides information on the associated complication and the natural course of the condition. The ophthalmologists and the researchers are not certain about the pathogenesis and the natural course of MF [2,3,4]. This might be an early stage of retinal detachment, secondary to a macular hole [5,6].

It was noticed that in highly myopic eyes, a progressive axial elongation occurs. This causes significant posterior scleral elongation and creates shearing forces within the eye and its internal structures: choroid, retina, and vitreous body. The result of this progressive stretching is the failure of these structures, which can cause known myopic maculopathies, such as chorioretinal atrophy, breaks in Bruch membrane, foveoschisis, macular hole, and, finally, retinal detachment. All these myopic maculopathies have, as a result, the deterioration or loss of vision.

The aim of the treatment is to release both the traction caused by the posterior staphyloma (the abnormal elongation of the sclera) and the anteroposterior traction caused by the vitreous cortex. One way of treating the condition is by entering the eye with wide viewing systems and working inside the eye. The vitrectomy is called the surgical technique of vitreous removal. The second step is peeling the epiretinal membrane and the internal limiting membrane, tissues in the surface of the retina. The aim of this second step is to release the tangential traction, the traction in the surface of the retina, at the area of the macula. It seems, however, that by performing only these surgical steps, only a transient release of the traction is achieved [7,8].

Ophthalmic surgeons have thought of proceeding with, in a third step, the reshaping of the posterior scleral wall by means of macular buckling. Reshaping the posterior scleral wall at the macula area, using a buckle, corrects the disproportion between the retina and the elongated sclera [9]. This way of treatment addresses the posterior staphyloma, the abnormal elongation of the sclera, which is the major risk factor associated with the development of macular foveoschisis.

Additionally, the macular buckling pushes closer the Retinal Pigment Epithelium (RPE) tissue, the feeding layer of the retina, to the photoreceptors, the light-sensitive layer of the retina. The final outcome is the reinforcement of the weak adherence between the RPE and the neurosensory retina because of the severe myopic chorioretinal atrophy [10,11]. A satisfactory anatomic, as well as functional, result is achieved with this procedure [12,13]. Another reason which favors macular buckling is the fact that the vitrectomy result takes a long time to achieve its anatomic goal. Macular buckling, on the other hand, has a significantly shorter period of recovery [14]. The two techniques can also be combined in order to achieve the best approach and the optimal final outcome.

The surgical technique starts with 360 degrees conjunctival peritomy and continues with placing sutures around the ophthalmic muscles in order to be able to move them towards the direction the surgeon wishes. The next step is to place the macular buckle, sliding it towards the macula. Special care must be taken during the placement in order to avoid traumatic damage to the eye vessels [15].

Many different macular buckles have been designed in order to create the best buckling device [16]. Many of them have been abandoned because of the difficulty of placing sutures in order to secure them. Others have been abandoned because they caused serious complications during manipulation, and yet others because it was difficult to monitor them intraoperatively. To resolve all these problems, some modifications have been designed. Intraoperative monitoring is achieved with the placement of a light pipe in a canal, all the way up to the end of the buckling area. This, in combination with the high magnification guaranteed with the new viewing systems, has allowed surgeons to monitor the course of the buckling, avoid traumatic complications, and place the buckle at the optimal position, under the macula. The optimal placement leads to a satisfactory anatomical and functional result.

The aim of this research is to design a buckle that perfectly fits the patient’s eye in each medical case. The buckle should be easy to use, with minimal need for manipulation, easy to monitor intraoperatively, and easy to place under the macula. A methodology for producing personalized tailor-made macular buckles for surgically treating myopic foveoschisis is developed and presented. The macular buckle, in this case, aims to restore this abnormality by mimicking normal human eye anatomy. This is achieved by developing an exact replica of its Three-Dimensional (3D) geometry on the computer and in a physical mockup with 3D printing technologies and biocompatible polymers. The main focus of this work is the development of macular buckle 3D geometry that is customized and fitted, in a better way, to each patient’s eye. 3D printing was employed for the manufacturing of the customized macular buckle since it provides the ability to produce complex geometries with biocompatible materials in a very short amount of time, with time being an important parameter in this case since the surgical treatment should be performed in a timely manner after the diagnosis and should not be affected by the required time for the manufacturing of the customized implant. Hence, the methodology presented herein was optimized for using 3D printing as the manufacturing method of the macular buckle.

With this methodology, the implant is individually designed according to the specific geometry of each patient’s eye, providing a more accurate solution than the commercially available generic implants. This tailor-made implant can be manufactured with various production methods. In this work, the Fused Filament Fabrication (FFF) 3D-printing method was employed since it is a fast and automated process that can nowadays produce parts with biocompatible polymers, such as PolyEther Ether Ketone (PEEK).

## 2. Materials and Methods

**Figure 1:**
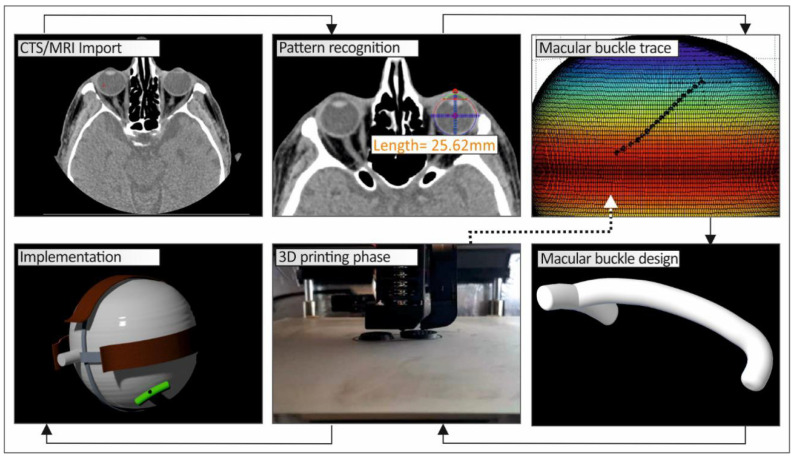
Flow chart describing the methodology developed to produce a personalized macular buckle.

Figure 1 shows a schematic graph of the methodology developed in this work to produce a personalized macular buckle.

**Figure 2:**
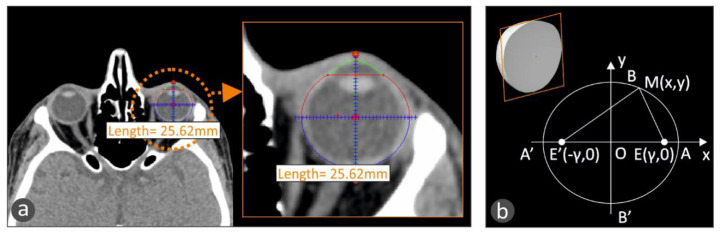
(**a**) Fitting an ellipse in the human eye circumference to more accurately capture the eye geometry in a CT scan image with the developed methodology. (**b**) The ellipse geometry that the human eye follows, according to human anatomy [16].

For the development of the tailor-made human eye implant, first, the patient’s eye geometry should be acquired with a medical imaging procedure. Computed Tomography (CT) was chosen, and the patient’s head was obtained primarily with this procedure in the form of medical images for each section taken during the process. The distance between the sections was chosen to be 1 mm. Top-down and front-to-back incisions were taken, and, consequently, there are images that do not contain the eye. With a software tool developed in Matlab Student (Mathworks, MA, USA), every tomography is checked manually, and if there is a part of the eye, the section is further processed. In the CT images that include the eye, the area that includes the eye was isolated. Then, pattern recognition algorithms were employed to isolate the perimeter of the eye in each CT image, which is in grayscale, and the circumference of the eye is selected and stored. In order to more accurately fit the geometry of the eye (Figure 2a), the assumption that each section is an ellipse, as human anatomy dictates [16], was made. An ellipse with focal points E’ (−*γ*, 0) and E(*γ*, 0), as shown in Figure 2b, is defined by the following equations:

(1) $$\frac{x^{2}}{\alpha^{2}}+\frac{y^{2}}{\beta^{2}}=1$$

(2) $$\mathrm{Where}\;\beta =\sqrt{\alpha^{2}-\gamma^{2}}$$

and AA’ = 2*α* and BB’ = 2*β*.

**Figure 3:**
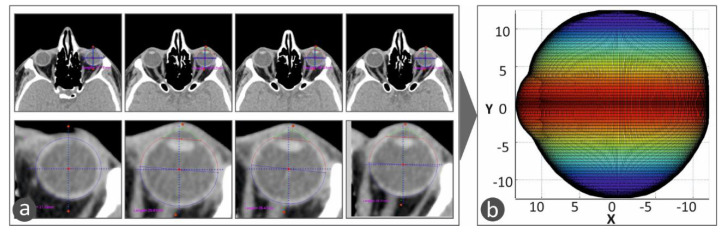
The 3D model of the human eye in the developed software. A color scale was used in the vertical direction to show the human eye height vs. the change in the human eye section size in the horizontal direction: (**a**) CT scan images in which the perimeter of the eye is determined and acquired, (**b**) 3D geometric model of the eye determined from the CT scan images and represented with a color scale

These algorithms were developed, and the CT images were processed in the Matlab Student software (Mathworks, MA, USA), which provides integrated tools and flexibility in reading and editing the tomography images. All the CT images were scanned, one by one, and the perimeter of the specific eye was acquired from each slice. The algorithm considers any movement of the patient or the patient’s eye during the scan in order to avoid developing distorted geometric models during the procedure to capture the human anatomy of each patient. Taking into consideration the slice thickness, the space between slices, and the pixel spacing, a three-dimensional geometric model of the eye was developed. The three-dimensional representation of the eye shows the iris and the quadrants of the eye. This representation of the 3D geometry helps the operator position the macular buckle accurately (Figure 3). Figure 3a shows typical CT images, in which the perimeter of the eye is determined and acquired for the determination of the 3D geometry of the eye. Figure 3b shows the 3D geometric model determined from the CT scan images and represented with a color scale. The color scale at this face of the work was used in the vertical direction to show the human eye height vs. the change in the human eye section size in the horizontal direction.

**Figure 4:**
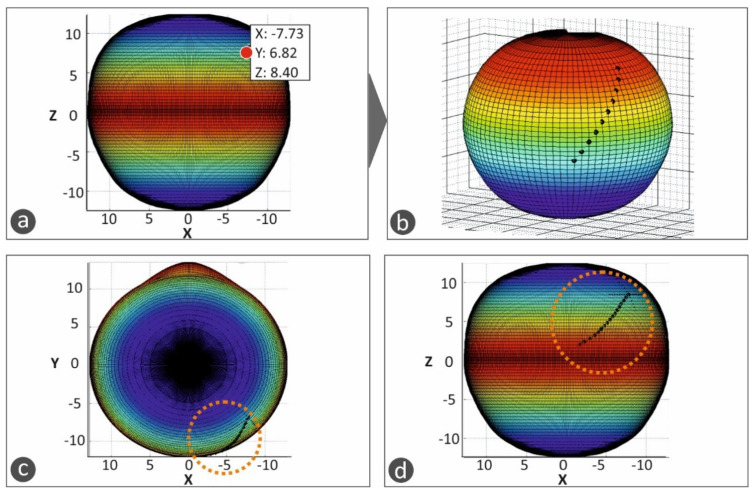
The procedure for the definition of the macular buckle implant trajectory and shape in the software developed in the current work: (**a**–**d**) show the procedure for the determination of the trajectory the macular buckle geometry should follow. Points are defined by the user in the macula position in the eye and the buckle geometry is defined according to the path defined by these points in space selected in the outer surface of the eye.

This 3D geometric model of the patient’s eye was employed for the development of a macular buckle implant that fits accurately to the specific eye’s geometry. Based on the results presented in the literature [17], a T-shaped macular buckle was selected. A software tool was developed in which the user selects the desirable starting and ending points of the implant in the outer surface of the eye’s geometry. This defines a path on the outer surface of the eye that the macular buckle will follow. Then, the point of interest of the macular buckle is selected on this path, and, finally, the required pressure that the macular buckle should apply to the eye is also selected (Figure 4). Figure 4a–d show the procedure for the determination of the trajectory the macular buckle geometry should follow. Points are defined by the user in the macula position in the eye and the buckle geometry is defined according to the path defined by these points in space selected in the outer surface of the eye. The macula position was determined according to the literature (2.5 times the optic disk diameter) [18].

**Figure 5:**
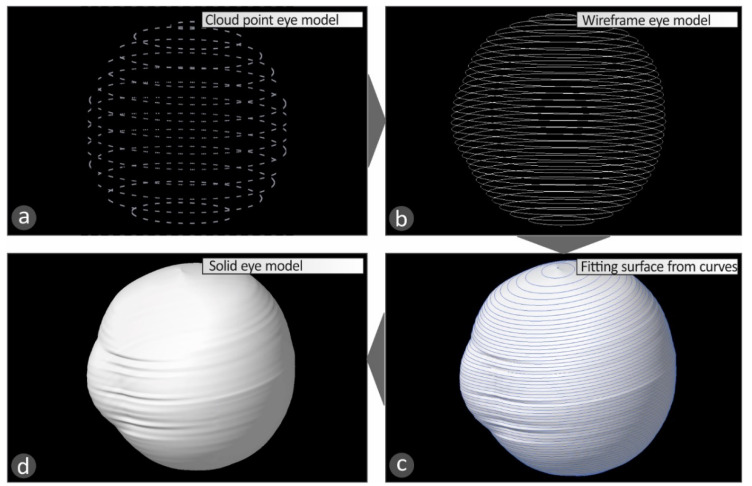
Steps for the development of the 3D geometric model of the patient’s eye using a CAD software tool: (**a**) cloud points, (**b**) planar 3D curves, (**c**) fitting 3D surface to the successive curves, (**d**) the final 3D geometric model of the patient’s eye.

**Figure 6:**
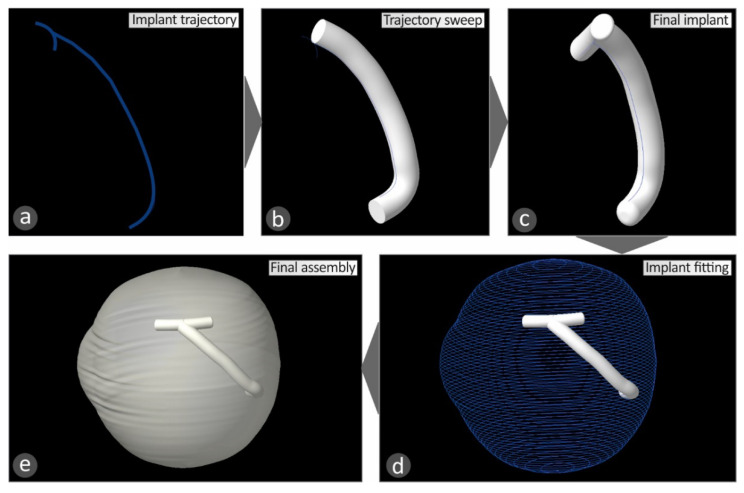
Steps for the development of the 3D geometric model of the customized macular buckle using a CAD software tool: (**a**) trajectory from the cloud points, (**b**,**c**) 3D curves, sweep geometry along the 3D curves, and finalizing of the implant design, (**d**,**e**) assembly of the human eye and the macular buckle using the CAD software tool to check the fit of the macular buckle to the specific human eye geometry.

Once the above procedure has been completed, the macular buckle technical parameters, i.e., the coordinates of the start and end points and the curve at the periphery of the eye, are stored, along with the human eye geometry, in an appropriate format (cloud points) for further use. By selecting the appropriate Computer Aided Design (CAD) software tool, the stored data are used to complete the construction of the three-dimensional geometric model of the patient’s eye (Figure 5) and the macular buckle, which is unique to that specific eye (Figure 6).

For the development of the 3D geometric model of the human eye, the cloud points acquired by the software developed on the Matlab Student (Mathworks, MA, USA) platform are imported to the CAD software tool. The points (Figure 5a) are converted to planar elliptic curves(Figure 5b), and these curves are then used to develop the 3D geometry of the human eye (Figure 5c,d). For the development of the macular buckle 3D geometric model, the cloud points acquired by the software developed on the Matlab Student (Mathworks, MA, USA) platform are imported in the CAD software tool. These points are employed for the design of a 3D curve (Figure 6a), defining the trajectory the implant should follow in the 3D space to fit the patient’s eye geometry. Then, the required shape the macular buckle should have is swept along this 3D curve to produce the 3D geometry of the macular buckle using the CAD software tool (Figure 6b,c). Figure 6d,e shows the assembly of the human eye and the macular buckle, using the CAD software tool to check the fit of the macular buckle to the specific human eye geometry.

The 3D geometric model of the macular buckle is then exported in a file format, suitable for use in 3D printers (stl file format), to be 3D-printed with a biocompatible polymer. In this work, the fused filament fabrication (FFF) 3D-printing process was used. It provides the ability to produce parts with biocompatible polymers such as polyether ether ketone (PEEK), which can additionally be sterilized with a steam device at 121–134 °C [19]. More specifically, an Intamsys Funmat HT (Shanghai, China) 3D printer was used, and the macular buckle was 3D-printed using PEEK polymer, with 100% solid infill, 45 degrees deposition orientation angle, 0.2 mm layer height, 390 °C 3D-printing nozzle temperature, 155 °C heat bed temperature, and 85 °C 3D-printer chamber temperature. In order to verify the fitting of the 3D-printed macular buckle, prior to the surgery, the 3D geometric models of the patient’s eye were also 3D-printed with the same 3D-printing parameters using polylactic acid (PLA) polymer (a biocompatible polymer).

The macular buckle will be surgically placed outside the sclera, at the point where the retina is detached. At this point, it pushes the sclera into the eye, thereby reconnecting the retina to the sclera. For the purposes of this study, only prototypes were developed, and the developed biocompatible implants need to be medically approved prior to their use in medical cases.

## 3. Results

**Figure 7:**
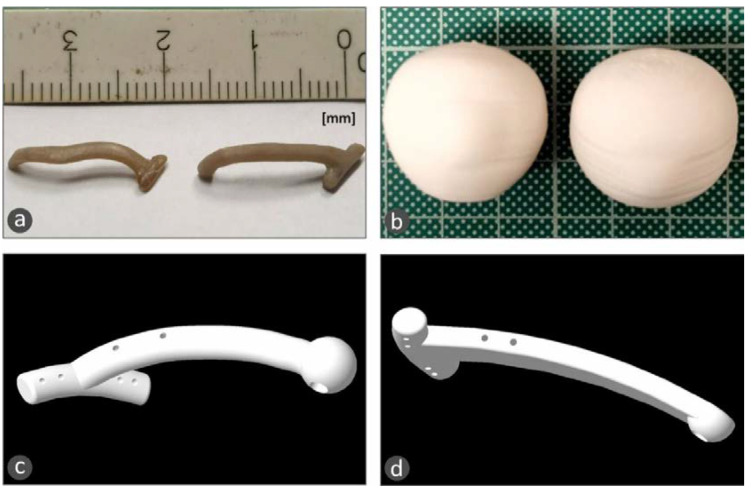
(**a**) 3D-printed customized macular buckle with polyether ether ketone (PEEK) material; (**b**) 3D-printed patient’s eye models with polylactic acid (PLA) material; (**c**,**d**) typical shapes of the prototypes developed for the macular buckle after the designed optimization proposed by the medical team of this work.

Figure 7a shows two different macular buckle implant 3D-printed prototypes manufactured with the developed methodology of the study using medical-grade PEEK biocompatible polymer, while Figure 7b shows the patient’s eye models that were 3D-printed with polylactic acid (PLA) polymer, which is also a biocompatible polymer.

The prototypes were evaluated by the medical team of this work, and design optimization was proposed in order to increase the functionality of the implant. A tunnel was opened throughout the implant for the insertion of the light pipe (with a stop feature at the end of the tunnel). The use of a light pipe is obligatory during the operation in order to get the best positioning, with fewer maneuvers. With the proposed design, the optimum lighting, which is crucial during the surgery, is achieved at the place where the exoplant is fixed in the eye. Additionally, slots were opened for the fixing of the implant with medical sutures in the horizontal and vertical parts of the T-shaped implant. The end of the vertical part of the T-shaped implant was redesigned to have different shapes, such as spherical or semispherical. Finally, the main body of the T-shape implant can also have different shapes, such as circular or rectangular, according to the specific needs of each case. Typical shapes of the prototypes developed are shown in Figure 7c,d.

## 4. Discussion

In this work, the aim is to develop the perfect buckling design that makes the surgical procedure easier and safer, with the need for less extraocular muscle manipulation, less manipulation of the staphyloma, and fewer scleral sutures. This means fewer complications in a difficult surgical procedure. A personalized implant will help the surgeon achieve the placement of the exoplant under the macular area, avoiding unnecessary manipulations. An exoplant model was designed, aiming to avoid all the reasons that caused rejections in the past. It is customized in order to achieve minimal manipulation and less damage to the vessels. Holes are placed in order to pass sutures and achieve good stabilization in certain key places of the exoplant. A canal was predesigned in order to place a light pipe for easy guidance.

The disadvantage of the novel implant is that a CT scan is required in order to produce the most optimized buckle. In the future, the aim is to use a method in which radiation will be avoided by the patient by using lasers or b-scan parameters to achieve the same result. The authors are convinced that a good model of the macular buckle can prevent all the long-term complications linked to the progressive worsening of staphyloma, such as atrophy of the retinal pigment epithelium.

In this work, a methodology for producing tailor-made macular buckles for surgically treating myopic foveoschisis is developed and presented. With this methodology, the implant is designed according to the specific geometry of each patient’s eye, providing a more accurate solution than the commercially available generic implants. This individualized implant can be manufactured with various production methods. In this work, the fused filament fabrication (FFF) 3D-printing method was employed since it is a fast and automated process that can, nowadays, produce parts with biocompatible polymers such as polyether ether ketone (PEEK). The authors hope that the macular buckling technique developed in this work will show very promising results, both anatomically and functionally, when it enters clinical trials.

Apart from this change, the methodology needs to be optimized for industrial applications. The development of both the patient’s eye geometry and the customized implant geometry needs to be more automated in order to be optimized.

Regarding the macular buckle, its design needs to be further optimized for increased durability as 3D printing is a fast and suitable method for its production and the 3D-printed parts have inferior mechanical strength when compared to injection-molded parts. Finally, in order to apply the developed methodology in medical cases, the macular buckles developed with this methodology need to comply with all the relevant medical standards, so all the related medical approvements need to be acquired prior to the introduction of the customized macular buckles to patients.

## 5. Conclusions

A lot of research has been conducted for the treatment of the macular foveoschisis complication, with most works showing that the most promising method is the surgical approach for the placement of a macular buckle implant that restores eye positioning and functionality and the human anatomy in this region in general. The existing commercial implants have a generic geometry, and although efficient enough, their placement during the surgery requires a lot of manipulation from the surgeon. In this work, the aim was to develop a macular buckle that fits perfectly in each specific patient’s eye and requires minimal manipulation during the surgery to be placed in the macula. Additionally, its geometry is expected to function in a better way, achieving improved overall results in the treatment of this complication.

To achieve this, a methodology was developed and implemented for the development of a tailor-made macular buckle according to the exact and specific geometry of the patient’s eye in each case. First, the geometry of the patient’s eye is captured and replicated in a computer program and then the macular buckle that perfectly fits this specific geometry is developed and manufactured. In this work, 3D printing with PEEK biocompatible polymer was used for the manufacturing of the implant, a method that is fast, accurate, and economically efficient for such applications.

The methodology presented needs to be further optimized for industrial and clinical use and needs to meet medical and clinical standards before its application in clinical settings. It is expected that when it complies with the medical standards, it will significantly improve the surgical procedure and the treatment of this complication, improving the patient’s life.
